# When it comes to stopping tuberculosis, what is actually “missing”?

**DOI:** 10.1371/journal.pgph.0000319

**Published:** 2022-03-23

**Authors:** Farahdiba Zalika Fatah, Jennifer Furin, Madhukar Pai

**Affiliations:** 1 Indonesian Young Coalition for Tuberculosis (IMUT), and Yarsi University, Jakarta, Indonesia; 2 Department of Global Health and Social Medicine, Harvard Medical School, Boston, MA, United States of America; 3 School of Population and Global Health, and McGill International TB Centre, McGill University, Montreal, Canada; Stellenbosch University, SOUTH AFRICA

While the world’s attention has been transfixed on the deadly, airborne COVID-19 pandemic due to the SARS COV-2 virus, another crisis has been unfolding when it comes to an ancient, deadly respiratory disease—tuberculosis (TB). Long ranked as the leading infectious killer of adults worldwide—and now second only to COVID-19—the tenuous gains that seemed to be won in the fight against TB have been disastrously undone in the past two years [1].

For the first time in more than a decade, deaths from TB—a completely preventable and curable infection—have risen [2]. Although the blame for some of the increased mortality is no doubt due to the socioeconomic hardships faced by people during the global lockdowns, much of it is also due to the reductions in TB diagnosis and treatment initiations that happened when COVID-19 hit the already tenuous health care systems that are all-too-common in high-burden TB settings [1]. Of the estimated 10 million people who became sick with TB in 2020, for example, only 5.8 million were given a diagnosis and started on treatment, a drop-off of nearly two million from 2019 figures [2].

Although COVID-19 has certainly made the global TB situation more dire, this age-old disease has long been starved of investments and innovations [3]. COVID-19 merely worsened an already neglected area of global health. The fact that we just last year ‘celebrated’ the 100^th^ anniversary of widely used anti-TB BCG vaccine (shockingly still the best preventive tool we have in the TB armamentarium) underlines how neglected TB product development has been [3,4].

When trying to excuse the dismal efforts at TB control that have characterized the field, much is made of the individuals who are sick with TB but who do not make it into the official counts of people who are diagnosed or treated for the disease. Referring to these men, women, and children as the “missing millions”, numerous global campaigns have been launched to “find the missing millions” over the last decade [5,6]. Despite this, not a single global target set forth in the 2018 United Nations High-Level Meeting on TB is on track to be met [2].

Instead of looking for people who are ‘missing’, it may be time for the global TB community, including us, to ask instead what is really missing in our TB response? Why are we failing people who are sick and scared and suffering, and why are they not being served by the institutions that are responsible for accompanying them during this grueling phase of their lives? What are the real reasons for people not seeking care in a timely manner? What access barriers do they face? Are people ‘hard to reach’ or health services hard to reach?

We believe there is nothing about people or what they are going through that is missing in any way. Rather, it is instead the global TB community that might be guilty of doing most of the missing when it comes to a comprehensive approach to ending TB [7].

We can see many areas where the TB community is missing. To begin with, we have missed the chance to take lead from TB survivors and affected communities. While the HIV/AIDS community has placed people with HIV at the center of their response, the TB field has mostly taken lead from medical and technical experts [8]. The fire that has driven other activist-led approaches to infectious diseases (e.g. HIV/AIDS) is subdued in TB, and could use greater dynamism. The TB field must therefore work harder to be of humble service to all people affected by TB. The TB field has largely chosen the path of non-confrontational and tokenistic compromises, since people with TB have never been prioritized, or requested to make ‘polite’ demands.

The TB community is yet to make the investments needed to prioritize wide-reaching and continuous health promotion to improve knowledge, attitude, and behaviour of communities affected by TB. We are yet to scale up the modern tools to offer easy diagnosis and curative treatment for people with all forms of TB. We are still using vaccines (i.e. BCG) and tests (i.e. microscopy) that are more than a century old and toxic drugs that must be given as part of months-long therapies [9].

People with TB ask for people-centered and humane services [10]. TB programs have instead pushed the need for a “one-size fits all” model of care delivered via a punitive and distrustful system (e.g. direct observation of treatment). People with TB have asked for more counseling and mental health services [10]. Most TB programs are yet to deliver such help to people with TB and those around them who are affected.

People with TB have also demanded for a tailored approach in designing products as well as interventions from the perspectives of key and vulnerable populations, especially those who face barriers to access healthcare [10]. For example, they have pointed out the challenges of toxic, injectable drugs that can cause deafness and other adverse effects, and have demanded all-oral, shorter drug regimens.

Also missing is the leadership needed to move beyond platitudes, patriarchy and institutional protection to focus on the transformative ideas needed to make lasting change [11]. Good leadership includes demanding the funding that is needed to actually address TB rather than settling for the paltry sums that are offered. For example, TB research-and-development investments reached only $0.9 billion in 2020, as compared with a need of $2 billion, estimated by *Global Plan to End TB*, *2018–2022* [1*]*. Given this under-investment, it is not shocking that we are still using century-old tools such as BCG vaccine and sputum smear microscopy.

Equity and social protection should be the core of all TB activities [12], but we fall back instead on colonial models of care dressed up in politically correct terms like “feasibility” and “sustainability” and “cost-effectiveness” [13]. While we have the chance to stop this disease in its tracks once and for all with a comprehensive approach, we have opted instead for piecemeal or incremental solutions in which TB seems to thrive [7].

As we mark yet another World TB Day on 24^th^ March characterized by catchy phrases that belie how badly we are failing when it comes to this disease, perhaps we should revisit the “finding the missing” rhetoric from a few years ago. But instead of pointing fingers at people affected by TB—who may be missing meals and missing work and missing family members who died from this travesty of a pandemic but who are most certainly not themselves missing—we should instead focus on what is actually missing when it comes to ending TB–this includes a more dynamic, empowered civil society, stronger leadership, greater investments, modern tools, and a more comprehensive, humane response.

Such an unflinching assessment would likely reveal that the blame lies squarely with the global public health community and our failure to do what has been needed for so many decades. Letting another year pass without such a reckoning will be once again missing the point.
